# Using design of experiments to guide genetic optimization of engineered metabolic pathways

**DOI:** 10.1093/jimb/kuae010

**Published:** 2024-03-15

**Authors:** Seonyun Moon, Anna Saboe, Michael J Smanski

**Affiliations:** Department of Biochemistry, Molecular Biology, and Biophysics, University of Minnesota, St Paul, MN 55108, USA; Biotechnology Institute, University of Minnesota, St Paul, MN 55108, USA; Biotechnology Institute, University of Minnesota, St Paul, MN 55108, USA; Department of Biochemistry, Molecular Biology, and Biophysics, University of Minnesota, St Paul, MN 55108, USA; Biotechnology Institute, University of Minnesota, St Paul, MN 55108, USA

**Keywords:** Metabolic engineering, Design of experiments, Gene expression

## Abstract

Design of experiments (DoE) is a term used to describe the application of statistical approaches to interrogate the impact of many variables on the performance of a multivariate system. It is commonly used for process optimization in fields such as chemical engineering and material science. Recent advances in the ability to quantitatively control the expression of genes in biological systems open up the possibility to apply DoE for genetic optimization. In this review targeted to genetic and metabolic engineers, we introduce several approaches in DoE at a high level and describe instances wherein these were applied to interrogate or optimize engineered genetic systems. We discuss the challenges of applying DoE and propose strategies to mitigate these challenges.

**One-Sentence Summary:**

This is a review of literature related to applying Design of Experiments for genetic optimization.

## Graphical Abstract

**Graphical Abstract:**
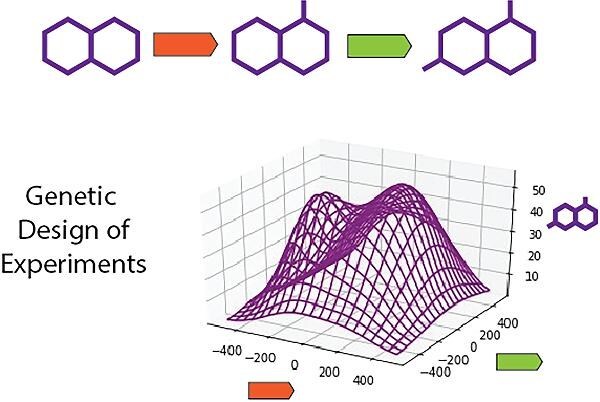

## Introduction

The sentiment of “the more, the merrier” does not often hold true when building and testing alternative genetic designs for a metabolic engineering project. Finding the best genetic design is a combinatorial optimization problem, with several relevant variables. These include (i) the source of enzyme-encoding DNA sequences (CDSs), (ii) the *cis*-regulatory elements (e.g. promoters, ribosome-binding sites [RBSs], terminators) controlling the strength of expression for these CDSs, (iii) the order and orientation of multiple cistronic units in a multigene system, and (iv) myriad environmental variables that can impact behavior/activity of these genetic designs in the host cell. As the number of genes encoded in a designed metabolic pathway increases, the size of the total genetic design space quickly becomes intractable. For example, designing an eight-gene pathway with just three different combinations of *cis*-regulatory elements per gene would have 3^8^ = 6561 possible designs (assuming that gene order or direction is not varied in the design space!). Increasing that number to 28 genes, as would be required to synthesize vitamin B_12_ in *Escherichia coli*, would mean that there are 3^28^ or 2.3 × 10^13^ possible sequences (Fang et al., 2018). Developing reliable and reproducible strategies for navigating this intractably large design space during genetic optimization projects is important.

A common strategy to address this problem is one-factor-at-a-time (OFAT) optimization. This involves altering one variable while keeping the others constant. The goal is to find the setting for the altered variable resulting in the highest yield of the end product. After optimizing one variable, that value is made constant as the next variable is optimized (Frey et al., 2003). Subsequently, one can continue moving in a stepwise fashion from one variable to the next, setting a new baseline each time a variable is optimized. This method has been used frequently to optimize fermentation cultures by varying nutrient levels, sources of nutrients, or culture conditions such as pH, oxygenation, temperature, and incubation time (Kheiralla et al., 2018; Singh et al., 2011; Sreena & Sebastian, 2018; Tan et al., 2011).

While this strategy can result in multifold improvements of product yield, there are several downsides. It is time and resource intensive due to the extensive number of experimental iterations required. This increases as the size of the system grows. Additionally, for systems in which the variables are not perfectly independent, the final combination of variable set points after an OFAT approach is likely to be suboptimal (Weissman & Anderson, 2015). The degree of suboptimality will depend on the order in which variables were perturbed.

An alternative to the OFAT approach is to use multivariate experimental analyses in the form of Design of experiments (DoE). Design of experiments is defined as a statistical modeling strategy that can be used to plan and analyze experiments. It allows for the simultaneous analysis of multiple variables, otherwise referred to as factors. This strategy is beneficial for determining how different elements of an experimental system impact one another. Understanding interactions among factors further helps avoid getting trapped in suboptimal local maxima (Heinsch et al., 2018). This process has been used with pharmaceutical drug development, media optimization, manufacturing processes, and bioremediation among other types of experiments (Islam et al., 2009; Kasemiire et al., 2021; Mandenius & Brundin, 2008; Rekab & Shaikh, 2005).

In DoE, variables play a pivotal role in shaping the experimental design and analysis process and can be broadly classified into two main types: categorical and continuous. Categorical variables delineate qualitative attributes into distinct groups. Nominal and ordinal variables are two subtypes of categorical variables. Nominal variables represent categories without inherent ranking, such as strain types, promoter types (i.e. constitutive, inducible, repressible, etc.), or media components (i.e. carbon source, nitrogen source). Ordinal variables, on the other hand, show a specific order or ranking but lack consistent intervals between categories, evident in order of genes in the gene cluster. In contrast, continuous variables provide quantitative measurements with infinite values within a defined range, including parameters such as pH, temperature, weight, or even strength of regulatory elements. Recent advances in the design and measurement of promoter and RBS strengths allow these factors to be considered as continuous variables, rather than as ordinal values. Promoter and RBS strengths can be quantitatively characterized using various experimental techniques, such as reporter assays and fluorescence measurements. These techniques provide quantitative measurements of gene expression levels under different promoter and RBS configurations, allowing for the assessment of their relative strengths. The Endy group was able to perform relative activity measurements using an in vivo reference standard, which will facilitate the measurement of promoter activities in multiple laboratories (Kelly et al., 2009).

During a DoE experiment, these biological and physical factors of interest are discretized into a set of values that are referred to as levels. These levels are then tested in different combinations, and a model predicting the response of the system is made based on input data and data gathered through many iterations of experimentation. DoE can be used in different ways to explore the effect of factors on a system. First, it can help define the range of levels that each factor should be tested at, which results in the total “design space” of the system that is then used later for optimization. It can also be used to test a large number of factors and determine a smaller number that are most impactful to the system. Finally, DoE can be used to optimize a system by looking at a smaller subset of factors that have been deemed to be most important (Weissman & Anderson, 2015).

We propose that DoE is a tool that should be routinely implemented in metabolic engineering applications. It provides the opportunity to optimize multiple design and experimental factors simultaneously, reduces the number of experimental iterations, and accounts for the importance of variables that may not have been considered noteworthy in other approaches. This is of particular importance to biological systems that have a large number of possible combinatorial designs. In this review, we will give examples of the recent applications of DoE in the field of metabolic engineering and how they have helped optimize a variety of biological systems. We will also discuss the limitations with these approaches and the current hurdles with applying DoE for genetic optimization. Finally, we will examine how these limitations can be mitigated in the coming years to make DoE more efficient and practical for future metabolic engineering projects.

## Applications of DoE in Metabolic Engineering

**Fig. 1.**
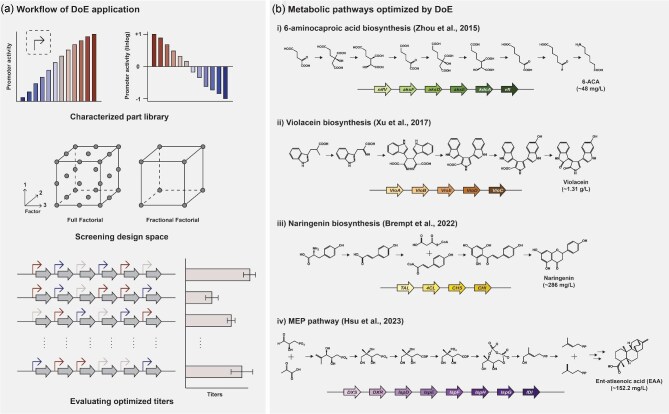
Design of experiments (DoE) application in metabolic engineering. (a) Schematic overview of key steps in the application of DoE, including characterizing genetic parts to provide quantitative control over gene expression variables, designing the survey of a multidimensional space, and evaluating system performance at the predefined points in that space. (b) Examples of pathways in which DoE has been applied to metabolic pathways.

As mentioned above, DoE can be used to answer many questions throughout a metabolic engineering project. Depending on the goal, different models can be used to plan experiments and optimize the genetics of an engineered metabolic pathway. Broadly, DoE can be classified as screening experiments or optimization experiments. Screening experiments seek to identify the variables most important for system performance, and optimization experiments seek to establish the optimal variable values. Most DoE methods are considered fractional factorial designs, as the total design space surveyed in the experiment is a fraction of the full factorial space (see Fig. 1). Fractional factorial designs are further divided into specific methodologies such as Plackett–Burman or regression models, which are commonly used in the field (Gündoğdu et al., 2016). Screening designs are primarily used to interrogate a large number of variables efficiently to find significant factors affecting the dependent variables that are further optimized by response surface methodology (RSM). Optimization designs use techniques such as RSM to refine a complex process through focusing on a small number of variables. Response surface methodology can also be further divided into separate methodologies, most often Box–Behnken design (BBD) and central composite design (CCD). Definitive screening designs (DSDs) are another type of fractional factorial designs that enable more efficient optimization processes as well as perform screening (Jones & Nachtsheim, 2011). Finally, machine learning is becoming more popular for use in optimization of experiments as an alternative to RSM, with one of the most notable forms being artificial neural networks.

### Screening Designs

*Full factorial designs*: Full factorial designs test every possible combination of factors in each of their discretized levels to generate a large and complete dataset of experiments. With full factorial designs, one can make an observation from each of these experiments and then perform statistical analysis to determine the magnitude of the effect of each factor as well as the interactions between factors (Cambray et al., 2018; Gündoğdu et al., 2016). This type of DoE has been used to understand the variables that affect translation efficiency in *E. coli* and to elucidate the nutrients affecting production of a recombinant protein aminolevulinate (ALA) synthase in *E. coli* (Cambray et al., 2018; Xie et al., 2003). The Altman group applied a full factorial design to investigate the impact of multiple nutrient factors on enzyme activity. A one-way analysis was used to estimate the ideal ranges for these factors. It was found that the initial concentrations of succinate, glucose, and Isopropyl β-d-1-thiogalactopyranoside (IPTG) were significant factors influencing ALA synthase activity. They used these results for a batch fermentation and achieved 10- and 18-fold higher ALA synthase activity and ALA titers, respectively, compared to previously reported results (Xie et al., 2003). The benefit of this approach is that it provides information about the nature of the interactions between factors. It may also reveal new interactions between variables that were not previously known, even in well-characterized systems. However, the downfall of full factorial designs is their costliness. Because so many experimental combinations are required, this method may not be best suited for researchers looking for a relatively fast and less resource-intensive way to optimize their system. Nor is it suited for large systems with many variables.

*Plackett–Burman fractional factorial designs*: Fractional factorial designs explore a relatively small number of possible variable combinations within the design space. This property makes fractional factorial designs better suited for screening systems too complex for a full-factorial design. Fractional factorial designs are used to identify the most important variables in a system and do not capture the full picture of interactions between factors. Plackett–Burman designs are fractional factorial designs used to categorize factors by their greatest level of importance (Hsu et al., 2023). Simple Plackett–Burman designs use two values (e.g. “high” and “low”) for each experimental variable. A Plackett–Burman matrix dictates which variable is in its high state and which variable is in its low state in each experimental trial. Plackett–Burman matrices have a special feature in that the effect of one variable on system performance can be considered in isolation with the effects of other variable canceling each other out. For example, in a simple two-state Plackett–Burman design that tests a seven-variable system (with variables A, B, …, G) using eight experimental trials, one could assess the impact of variable A by comparing the average performance of the four experiments that have A in its high state to the average performance of the four experiments that have A in its low state. In both of these subgroups, all of the other variables, B–G, are represented by exactly two high states and two low states. Importantly, the matrix is balanced in a way that this is true for any variable that is singled out to calculate its primary effect.

Fractional designs, including Plackett–Burman, are more suitable for experiments involving continuous variables compared to those with categorical variables. The quantitative difference between levels explored for each variable should be determined pragmatically. For more explanation, see the section titled “Choosing Appropriate Variable States.”

Plackett–Burman has been used to optimize the methylerythritol phosphate pathway in *Streptomyces* in addition to helping improve production of a violacein pathway introduced into *E. coli* (Hsu et al., 2023) (see Fig. 1; Xu et al., 2017). Our group investigated the use of a five-level Plackett–Burman fractional factorial design to enhance the titer of a heterologous terpene biosynthetic pathway in *Streptomyces*, particularly focusing on the production of diterpenoid ent-atiserenoic acid (eAA). Within the library of 125 engineered gene clusters, the eAA production titer exhibited significant variation, spanning over two orders of magnitude. They identified that the expression of dxs, the gene responsible for encoding the first and flux-controlling enzyme, had the most substantial impact on eAA titer through analysis of the Plackett–Burman design (Hsu et al., 2023). This system is beneficial because it exhibits balance and orthogonality, meaning that variables are tested equally in terms of frequency and any effect of other factors is mitigated to be able to focus on just one at a time (Hsu et al., 2023). A weakness of this approach is that the math underlying data analysis implicitly assumes that all variables are fully independent, which is often not true in biological systems. However, inclusion of internal controls, specifically as unassigned “dummy variables,” gives experimenters the ability to quantify the degree to which nonindependence of the system variables impacts the results. Operationally, Plackett–Burman designs have been proven effective in identifying high-impact variables in biological systems, including for medium optimization (Zhang et al., 2010) and metabolic pathway design (Hsu et al., 2023). This reflects the fact that the dependence/independence of variables exists on a spectrum. Systems for which the variables are not strictly independent can still have sufficiently small interacting terms, where the negative impact of these interactions of DoE conclusions is minor. These impacts introduce noise into the data analysis that can be mitigated by the application of statistics in a similar way that is true of any source of experimental variance or noise (Hsu et al., 2023).

*Fractional factorial regression modeling*: Another commonly used fractional factorial framework is regression modeling. Within regression modeling, one may use linear regression methods, including ordinary-least squares (OLS), or other regression models such as partial least squares (PLS) (Brempt et al., 2022; Brown et al., 2018; Lee et al., 2013; Singleton et al., 2019). Linear regression involves modeling the relationship between an independent variable and dependent variables. In such a test, the levels of the independent and dependent factors vary per experiment. A predicted value is generated by the model while an observed value is measured as a product of each experiment. In an OLS test specifically, the goal is to obtain a small sum of squared differences between the value of the predicted and measured experiments. This allows the model to generate a best-fit curve that represents the relationship between variables. In general, a smaller amount of variation between experiments indicates better accuracy of the response predicted by the model. This model is a simplistic method of analysis because it ignores the complex interactions that exist between biological factors. Lee et al. (2013) argue that this may be beneficial for biological systems since their complexities are hard to predict and therefore potentially beneficial to avoid altogether. To support this argument, their group demonstrated successful expression of the violacein biosynthetic pathway for the first time in yeast using this method (Lee et al., 2013). However, OLS assumes independence between variables such as Plackett–Burman and cannot capture the intricacies of biology that may be necessary to introduce a new metabolic pathway into an organism (Lee et al., 2013). Ordinary least-squares performance also decreases as the number of variables in a system increases due to the emergence of overfitting trends (Vinet & Zhedanov, 2011).

Partial least squares analysis is another form of regression modeling that may be a more useful model for biological systems. This method is different from OLS because it does not require the assumption of linear interaction between variables and is better able to handle the noise from biological systems (Brempt et al., 2022; Brown et al., 2018). The downfall of this method is that it is more susceptible to omitting important structural factors that correlate with a response in the system (i.e. Type II error) (Cramer et al., 2015). However, this is less likely to be detrimental to the optimization of a biological system than producing a list of many variables that are not in fact related to a response (i.e. Type I error). Partial least-squares is a method similar to principal component analysis. The purpose of PLS is to predict the changing response of variables as their levels are varied rather than detecting the influence different variables have on one another (Vinet & Zhedanov, 2011). Partial least squares models generate two matrices: one of manifest and one of response variables. The goal is to measure the covariance between these matrices to be able to pull latent variables (LVs) out of the system, which are attributed to generating the variation in response variables (Singleton et al., 2019; Vinet & Zhedanov, 2011). These LVs are not able to be directly measured but are inferred from the manifest variables that are measured. This method has been used to generate optimized chemically defined media, to confirm the understanding of the factors important to ethanol biosynthesis in yeast, and to optimize synthesis of naringenin in *E. coli* (Brempt et al., 2022; Brown et al., 2018; Singleton et al., 2019) (see Fig. 1). Ultimately, PLS is better at accounting for the nonlinearity of biological systems than other fractional factorial methods such as Plackett–Burman or OLS and therefore may be one of the best DoE methods for screening a complex metabolic engineering system exhibiting a greater degree of interconnectedness.

*Analysis of variance*: Analysis of variance (ANOVA) is another statistical technique in DoE used to analyze the variation in the experimental data (Sthle & Wold, 1989). ANOVA compares means across groups, while regression models estimate relationships between variables to predict outcomes. ANOVA will primarily be suitable for experiments dealing with categorical variables and tests for differences in means, assuming equal variances and normally distributed data within groups. Regression models, on the other hand, handle both categorical and continuous variables, estimating the effects of predictors on the outcome variable while assuming linearity and independence of observations.

The Voigt group has performed regression modeling and ANOVA analysis for biosynthesis optimization (Zhou et al., 2015) (see Fig. 1). They applied a fractional design to optimize a 6-aminocaproic acid (6-ACA) pathway in *E. coli*, comprising six heterologous enzymes. A 32-member fraction factorial library is created to simultaneously alter expression levels and media composition, compared to a 64-member full factorial library that varies only expression. Regression analysis was used to find the best construct within a specified range and ascertain the primary factors affecting the titer. The regression model predicted the genetic setup and media composition that maximizes 6-ACA production among the 512 potential combinations and also estimated that the response curve is quadratic polynomial from the data. An ANOVA analysis was conducted on the quadratic model to confirm its statistical significance. The *F* test, incorporated into the ANOVA analysis, assessed the statistical importance of each factor's impact on the final titer output. In the design of a two-level DoE library, a higher *F* ratio associated with a factor indicates that the difference in mean production titers between the high and low levels of that factor is significant. This suggests that altering this factor has a significant impact on the final production titer. Conversely, if there is negligible difference in titer between the two levels, the *F* ratio approaches 1. By applying a DoE approach with statistical analysis, they succeeded in boosting 6-ACA production from 9 to 48 mg/L.

### Optimization Designs

*Response surface methodology*: Aside from the full and fractional factorial screening designs listed above, optimization using DoE can also be performed with RSM. Response surface methodology differs from previously described DoE methods because the goal is to optimize response variables rather than finding the most significant variable. When using RSM, the most important factors of a system are already known and thus just need to be adjusted to their optimal levels in the system to achieve the most effective optimization result. The first popular form of RSM that is used for these experiments is CCD. These designs are five-level fractional factorial designs that require more experiments than BBD (described below). These designs are beneficial for predicting linear, quadratic, and interaction effect variables in a system, and typically provide lower residual standard error values than BBD (Gündoğdu et al., 2016). CCDs contain center points in the middle of the experimental space where factors are set to the mean value of their range of levels. These center points can be surrounded by cube and star points. Cube points are discretized to the levels of each factor representing either +1 or −1 from the value of the center point. Star points are discretized to the minimum and maximum of each factor that lay outside of a theoretical cube representing the design space, thus transforming the shape of the design space into a sphere (Bevilacqua et al., 2010). It is beneficial for CCD systems to be rotatable, meaning that the levels of each factor could be put on a circumference and rotated in order to best perform with quadratic polynomial problems (Bevilacqua et al., 2010; Gündoğdu et al., 2016). This method of DoE is beneficial for sequential experiments because you can add onto a design by adding more cube and star points (Bhattacharya, 2021). CCD has been used to optimize production of cellulase in *Bacillus subtilis* after OFAT and Plackett−Burman were used to determine the most important factors in the system (Sreena & Sebastian, 2018). Their OFAT analysis determined the optimal incubation temperature and agitation speed (150 rpm), and their Plackett−Burman design also screened significant variables influencing cellulase production, identifying carboxymethyl cellulose (CMC), yeast extract, NaCl, pH, MgSO4, and NaNO3 as most influential, with CMC, yeast extract, NaCl, and pH showing positive effects. Following RSM via CCD determined optimal levels of CMC (13.46 g/L), yeast extract (8.38 g/L), and NaCl (6.31 g/L) at pH 7, resulting in a maximum cellulase activity of 566.66 U/ml, close to the predicted activity of 541.05 U/ml. They found that this DoE optimization increased activity by 3.2-fold compared to unoptimized conditions (179.06 U/ml). Additionally, CCD has been used to optimize bacterial phytase production in *Pichia pastoris* (Akbarzadeh et al., 2015).

The second type of RSM is Box-Behnken design (BBD). BBD is useful for determining the quadratic effects between multiple interacting variables, building sequential designs, and detecting lack of fit in models (Islam et al., 2009; Xu et al., 2017). These models often employ a three-level system to their factors, meaning that each variable can be discretized into low, medium, and high levels (Gündoğdu et al., 2016). For some factors, every possibility is tested across the total number of experimental runs while only a handful of the total combinatorial possibilities are explored for others (Gündoğdu et al., 2016). This method is advantageous because it requires only a small number of variables to predict a complex and accurate response (Kumari & Gupta, 2019). Box–Behnken design is less resource intensive than CCD, requiring fewer experimental trials. However, BBD is not capable of detecting more than second-order interactions, which could potentially unveil important interactions between variables that could lead to even better optimization. Despite this limitation, BBD optimization has been used in the literature for a number of applications including optimization of the violacein pathway in *E. coli*, increased hydrogen yield in mixed batch anaerobic mesophilic cultures, and cellulose production in *Acetobacter xylinum* (Ray et al., 2010; Xu et al., 2017; Zeng et al., 2011). The Stephanopoulos group conducted fractional sampling of the gene expression landscape and identified statistically significant enzyme targets that impact the efficiency of metabolic pathways. They employed Plackett–Bruman for screening and BBD to apply empirical quadratic regression model to ascertain the most effective gene expression patterns for the pathway. They observed synthesis of violacein at 525.4 mg/L in shake flasks, which is a 3.2-fold enhancement over the baseline strain. They also mentioned that converting discrete gene expression levels into logarithmic variables was pivotal for executing this DoE-driven optimization approach (Xu et al., 2017) (see Fig. 1).

Therefore, this method is the most suitable style of RSM if one wishes to predict a response of a system when you do not need to know the response of extreme levels of a particular variable and is overall faster to use than CCD (Ray et al., 2010).

### Definitive Screening Designs

Definitive screening designs (DSDs) are a type of fractional factorial design and can estimate main effects as well as the interactions between factors that affect the response of the system. Unlike many other screening designs, DSDs are structured in a way that allows for the estimation of quadratic effects in addition to main effects and two-factor interactions. DSDs also help avoid the compounding of main effects with interactions between factors and pinpoint factors that exhibit nonlinear impacts on the response (Jones & Nachtsheim, 2011; Jones & Nachtsheim, 2017). DSDs also enable fit to a response surface model without doing additional experimental runs. Comparable in scale to factorial experiments, these trials offer a viable alternative when curvature is anticipated in the response variable. For example, you can accurately estimate main effects without being confounded by second-order effects with only 2 *N* + 1 of experimental runs for N factors (Jones & Nachtsheim, 2011, 2017; Walls et al., 2022). Moreover, using 6–12 factors can efficiently estimate all conceivable full quadratic models incorporating three or more active factors, which may be feasible to proceed to optimization without additional experimentation. If the number of active effects surpasses three, however, fitting the full quadratic model reliably becomes challenging (Jones & Nachtsheim, 2011, 2017; Walls et al., 2022). Overall, DSDs can be a cost-effective solution when you aim to both identify significant factors and optimize the response variable within a single set of experimental runs. This makes them an efficient choice for those focused on process optimization. The Rios-Solis group has used a three-level DSD to screen a range of defined media compositions to improve taxane production in *Saccharomyces cerevisiae* (Walls et al., 2022). They investigated the effect of six different factors, including initial cell densities, galactose concentrations, and other yeast media components, on activity of taxane-producing strain, LRS6. This group achieved at least two-fold improvement of taxane or taxol intermediates by applying a DSD.

### Machine Learning With Artificial Neural Networks

With the increase in popularity of machine learning for biological applications, strategies such as artificial neural networks (ANN) are being explored as an alternative to the statistical modeling methods listed above. ANN is beneficial because it can interpret nonlinearity and researchers can optimize a system without needing to know initial boundary conditions or important assumptions about different variables (Liyanaarachchi et al., 2020). By just training off of experimental data, there is no need to have predetermined knowledge about a system or the types of variables that may be most impactful to the system. This way, the problem of ignoring important factors in a system can be avoided. ANN models mathematically mimic the function of the human brain, and different connections are represented by artificial neurons. They involve input, hidden, and output factors, where the hidden factors help shape the understanding of the input and output variables. The links between neurons are referred to as weights, which represent the strength of the connection between neurons. These weights are determined while the model is being trained and help inform its predictive power for use in experimentation. The ANN networks give an idea for how interconnected the system is and can provide sound predictions for the best way to optimize a biological process. Overall, ANNs are beneficial over traditional RSM modeling because they can provide a smaller root-mean-squared error and prediction error, therefore indicating that they are more effective at modeling nonlinear systems (Liyanaarachchi et al., 2020; Sewsynker-Sukai et al., 2017). ANNs have been used to optimize cultivation conditions of microalgae for biodiesel production and to improve output of the violacein pathway in yeast (Liyanaarachchi et al., 2020; Zhou et al., 2018). The Kornaros group tested ANN models to predict the concentrations of biomass, total lipid, unsaturated lipid, and oleic acid in *Chlorella vulgaris* based on cultivation time and pH-level inputs. The predicted values demonstrated strong correlations with experimental data across all ANN models, outperforming RSM in prediction accuracy. Olden's plots revealed diverse influences of pH and time on different metabolites, underscoring the importance of careful optimization in *C. vulgaris* cultivation for biodiesel production. Through multiobjective optimization algorithms, the optimum cultivation time and pH for biodiesel production were determined. They also obtained improved optimal concentrations of oleic acid, biomass, total lipid, and unsaturated lipids (Liyanaarachchi et al., 2021). While ANNs have proven to be highly effective, one important consideration when using these algorithms is to ensure that high-quality data are being fed to the model when it is being trained; otherwise, that could impact the effectiveness of its optimization outputs (Brempt et al., 2022). Despite this potential obstacle, ANNs remain an effective tool that cuts down further on iterative experimentation in comparison to other fractional factorial designs or RSM and allows for avoidance of overfitting when performed with appropriate training and validation datasets.

### More DoE Methods for Future Applications

Many other experimental designs, though potent, have not yet been implemented in metabolic engineering endeavors. Below, we present some examples of noteworthy DoE methods poised for future application.

Several fractional factorial designs exist beyond Plackett–Burman. Cotter designs exemplify this, involving the division of experimental units into blocks or groups and the systematic assignment of treatments or conditions to each block in a balanced manner (Cotter, 1979). This strategy, termed “blocking,” will be further discussed in the “Blocking” section. The goal is to ensure that any sources of variation potentially impacting the experiment's outcome are evenly distributed across the different treatment groups. Cotter designs differ from Plackett–Burman designs in that they focus on the balanced assignment of treatments and controlling variability, while Plackett–Burman designs are optimized for the efficient screening of large numbers of factors in a fractional factorial design. Cotter designs prioritize reliability and robustness by accounting for sources of variation, whereas Plackett–Burman designs aim to identify main effects efficiently while conserving resources in screening experiments, particularly in the context of metabolic engineering.

Split plot designs can also be effectively used in metabolic engineering experiments where certain variables are difficult or costly to change (Jones & Nachtsheim, 2009). For example, in a metabolic engineering study aimed at optimizing the production of a biochemical compound in a microbial strain, factors such as genetic modifications and culture conditions may be considered as hard-to-change variables. These factors can be treated as main plot factors, while easily adjustable variables such as media components and fermentation parameters can be assigned as subplot factors. Split plot experiments are more practical for implementation in industrial settings by enabling experiments to proceed even in the presence of hard-to-change variables.

Taguchi array designs are other methods employed to identify signal factors while minimizing the influence of noise factors that are often challenging or expensive to control (Tsui, 1992). For instance, in the same study as above, Taguchi array methods can be used to systematically vary culture conditions (such as temperature, pH, and agitation rate) as signal factors, while noise factors (such as media composition variability) are accounted for using orthogonal arrays. This approach ensures output consistency by determining control factor settings that yield acceptable responses despite natural environmental and process variability.

## Limitations of Applying DoE for Metabolic Engineering

Several challenges impede the application of DoE to genetic system optimization. These include poor accuracy and precision in setting variable levels, measurement errors or experimental variability, and violations of some assumptions (e.g. variable independence) that underlie the design and interpretation of DoE datasets. While these limitations can decrease the effectiveness of DoE applications, their impacts can be mitigated with careful experimental design and data interpretation. Below, we discuss in more detail several of the most impactful limitations to applying DoE in genetic systems.

### Genetic Context Dependency

**Fig. 2.**
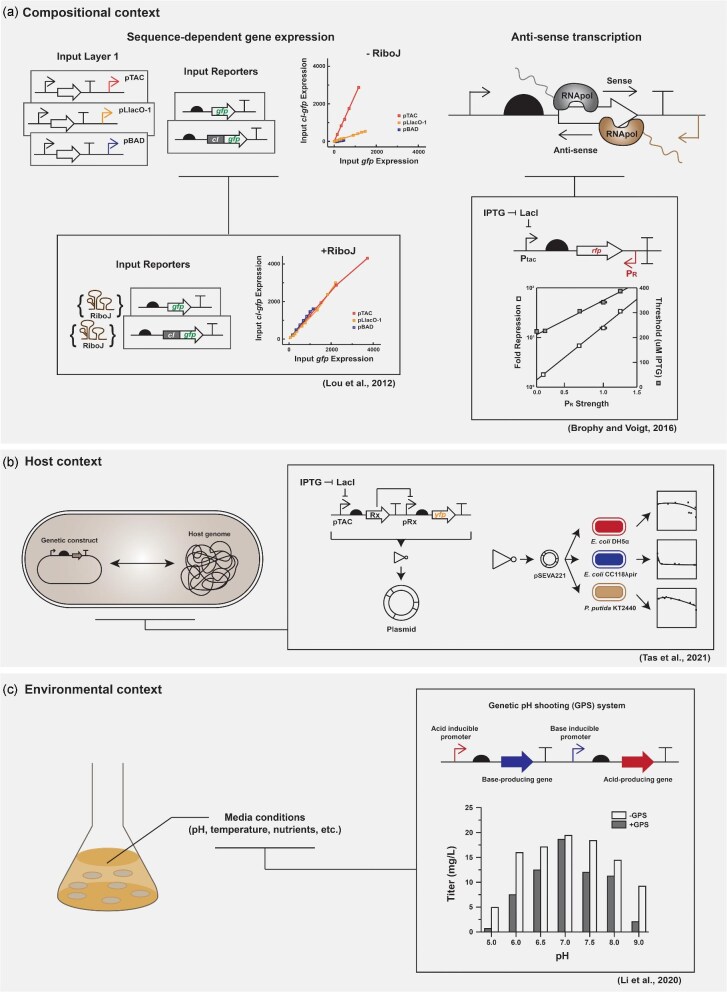
Approaches to overcome genetic context effects. Example studies that provide tools or frameworks to overcome compositional context effects (a), host context effects (b), or environmental context effects (c).

Genetic context dependency refers to the behavior of a genetic element varying as a function of its local environment. This is common in engineered genetic systems composed of many discrete functional genetic elements (e.g. promoters, CDSs, etc.). All genotypic changes are context-dependent, and this context governs the biological system in a complex and unpredictable manner. The main sources of context dependencies can be broadly classified into three types: compositional-, host-, and environmental-context effects (Cardinale & Arkin, 2012) (see Fig. 2).

*Compositional context*: Compositional genetic context effects arise from unpredictable physical or functional interactions between neighboring genetic elements. Physical composition problems may come from unwanted structural interaction of regulatory elements on the same DNA molecule. For example, swapping out a weak promoter for a strong promoter may change the 5′ untranslated region (UTR) in a way that unintentionally creates strong secondary structure and decreases translation initiation (Leppek et al., 2018). Context effects can occur not just from the physical interactions at the nucleic acid level but also if there are connections and interactions between the linked input and output processes. For example, differing substrate promiscuity or product profiles of homologous metabolic enzymes can impact their behavior in the context of a synthetic metabolic pathway.

*Host context*: Engineered genetic systems rely on the cellular machinery of host organisms for their expression and operation. The host machinery that processes DNA-encoded instructions varies slightly from host to host, leading to differences in promoter strength, RBS strength, translational efficiency, etc. Additionally, host-encoded proteins or RNAs can interfere with the engineered system by causing unwanted cross talk, leading to competition for targeted or designed molecular bindings or interactions (Engelmann et al., 2023). The engineered system may also consume limited resources or impose metabolic burdens that negatively affect the physiology of host species to different degrees, reducing cell fitness and ultimately decreasing the efficiency of the engineered system (Cardinale & Arkin, 2012).

*Environmental context*: Biological systems dynamically adapt to changing environmental conditions in real time to maintain a dynamic equilibrium. Therefore, the behavior of both the engineered system and the host organism is influenced by the environmental context, which consists of various factors such as temperature, pH, substrate concentrations, and more. These diverse environmental factors can directly affect the function or behavior of the components of the engineered system and indirectly influence the intracellular conditions of the host organism (Brophy & Voigt, 2014; Cardinale & Arkin, 2012).

Success of a DoE experiment hinges on the ability to set variables with accuracy and precision. For nonbiological applications in which DoE is commonly applied, this accuracy and precision is more easily attained. In genetic systems, context effects decrease accuracy and precision in variable settings. The ability to rationally tune a gene's expression level (and ultimately an enzyme's concentration in the cell) is fraught with relatively high levels of uncertainty. Strategies to mitigate this impact are discussed in more depth in the “Mitigating Risks to Improve DoE Robustness” section.

### Measurement System Maturity

DoE approaches rely on statistical analysis to determine the likelihood that observed patterns would be due to random chance. Many approaches, such as the Placket–Burman design, include dummy variables to serve as internal negative controls. As is true with any statistical analysis, large measurement error decreases the effect size that can be observed as statistically significant (Gent et al., 2018). Because of this, how well a particular DoE approach will work is highly influenced by measurement system maturity.

While biological systems will always have variance, experimenters should minimize the measurement variance that can be controlled as much as possible prior to beginning a DoE experiment. This can come from adopting best-practice standard operating procedures (Moreno-Camacho et al., 2019), employing process automation (Schmidt et al., 2022), and details concerning sample collection (Tworoger & Hankinson, 2006), including volume and timing of sample collection, are all known to impact measurement variance in biochemical experiments. Time and energy spent reducing measurement error will pay dividends by improving the confidence in results from a DoE approach.

### Unpredictability Arising From System Complexity

System complexity can hinder the effectiveness and accuracy of DoE approaches. One significant limitation lies in the assumption of independence within the models used. These models often assume that the variables under investigation are independent of each other, disregarding any potential interactions or dependencies that may exist. This assumption can lead to misleading predictions and hinder the understanding of complex relationships especially for biological systems that comprise highly interconnected networks. One could cite a DoE's assumption of variable independence to preclude its use in genetic optimization from the outset. However, it is important to acknowledge that variable dependence or independence exist on a spectrum. In between complete independence and complete codependence are varying levels of dependency, or strengths of interaction terms. DoE may prove relatively less effective for systems characterized by significant interaction terms between variables, especially when such interactions are not readily known or anticipated. However, despite the underlying assumption of variable independence in many DoE designs, they can work well for systems that display a modest degree of variable dependency. This has been empirically demonstrated in the literature (Hsu et al., 2023; Zhang et al., 2010; Zhou et al., 2015).

We have recently published a simulation study to explore the impact of system complexity and experimental noise on the performance of a Placket−Burman design. We simulated a variety of functions that fall in different regions on the spectrum of independent/complex systems. As expected, the ability of DoE to detect and return accurate predictions varied as a function of system complexity, measurement error, and accuracy in variable setting. Systems with multiple interacting variables never performed as well as independent variable systems that had low levels of measurement error or variable setting inaccuracy. However, they were also less sensitive to changes in those alternative sources of noise (Hsu et al., 2023).

## Mitigating Risks to Improve DoE Robustness

### Strategies for Genetic Context Dependency

Genetic context effects can pose challenges for genetic studies, but there are several methods that can be used to overcome these effects. Broadly, these can be categorized as strategies that either (i) leverage an understanding of context effects to rationally control gene expression levels or (ii) buffer against genetic context effects by employing alternative genetic design features.

*Leveraging genetic context effects*: Much has been learned about the impact of surrounding DNA sequences on the function or behavior of genetic elements. Within a single transcription unit, context effects between sequences that control transcription initiation rates (i.e. promoters) and translation initiation rates (i.e. RBSs) (Mutalik et al., 2013) can be at least partially mitigated through biophysical models based on first-principle understanding of nucleic acid interactions (LaFleur et al., 2022; Reis & Salis, 2020; Salis et al., 2009). These models allow for the specification of upstream and downstream nucleic acid sequences when making de novo quantitative predictions of the behavior of the genetic part of interest. In polycistronic transcription units, the order of coding DNA sequences impacts protein production in a predictable way (Lim et al., 2011).

Between multiple transcription units, the relative order and orientation of promoter sequences were found to impact their the transcription initiation rate (Smanski et al., 2014), due to the impact of local changes in positive or negative supercoiling that occur during transcription (Johnstone & Galloway, 2022) or collision of RNA polymerases (Brophy & Voigt, 2016) (see Fig. 2). This knowledge can be used to tune levels of gene expression in a more nuanced way than would be possible using only characterized expression levels from standardized reporter gene constructs.

Host genetic context effects exist even for subtle host changes, for example, switching between *E. coli* strains (Egbert & Klavins, 2012). Host-dependent changes in plasmid copy number and behavior of genetic NOT gates have been characterized in a combinatorial fashion to extend the range of functional Boolean logic gates that could be constructed (Tas et al., 2021) (see Fig. 2). These examples all point to a possible future wherein the causes and consequences of genetic context effects (even if understood only empirically but ideally if understood from first principles) could be factored into the design of genetic systems to predict their behavior more accurately. This would benefit the application of DoE by allowing a higher percentage of experimental designs to hit the desired variable set points.

*Buffering against genetic context effects*: Various genetic design approaches have been developed to buffer against genetic context effects and minimize their interference in the expression of engineered genetic systems. In regard to compositional context, these strategies typically involve the use of genetic insulators. Self-cleaving ribozymes, such as RiboJ, have been shown to buffer against unpredictable interactions between promoters and RBSs. By placing ribozymes between these expression elements, the 5′-end of a transcribed construct is standardized, regardless of which promoter was used to control its transcription (Lou et al., 2012) (see Fig. 2). The utilization of genetic safe harbor loci can provide stable genomic integration sites to mitigate effects related to the genomic context. Genetic safe harbor loci provide well-defined integration sites within a genome to introduce exogenous genetic material while minimizing disruptions to endogenous gene function (Ploessl et al., 2022).

Host context effects can be somewhat mitigated by using orthogonal transcription machinery, for example, T7 RNA polymerase to direct transcription of multiple transgenes (Liang et al., 2018; Temme et al., 2012). An orthogonal ribosome has been described (Aleksashin et al., 2020) but to our knowledge has not been ported between multiple organisms. Also, the orthogonal ribosome does not mitigate all sources of host genetic context, as tRNA availability and charge state would still be different in alternative hosts.

In order to minimize environmental context effects, Li et al. (2020) developed a genetic circuit that enables self-regulation of pH in a microbial cell factory, thereby reducing the impact of environmental factors on the system's performance (see Fig. 2). Additionally, the Bakshi group has created a valuable resource called the “context matrix.” This database serves as a conceptual framework for categorizing and exploring various genetic contexts and their collective effect on synthetic constructs. The context matrix enables decision-making regarding the important factors that influence the engineered biosystem's function and helps identify previously unknown factors for consideration (Moschner et al., 2022).

### Choosing Appropriate Variable States

As mentioned earlier, one requirement of an effective DoE is knowing the appropriate range over which to perturb your variables. This is complicated by the fact that multiple variables can be compared in a single experiment that do not have similar units of measure (e.g. concentration of glucose in the growth medium [g/L] and expression level of a biosynthetic gene [relative expression units]). Setting the variables across too large of a range can decrease the possible information content in the experiment. In an extreme example, if in a two-level Plackett–Burman experiment the pH is varied at either pH = 1 or pH = 7, it is highly likely that the inappropriately low set point of 1 would diminish the effectiveness of the analysis. Half of the cultures would have no growth/viability. Setting the range too narrowly (e.g. relative expression level of 100 vs. 105 arbitrary units for a gene of interest) is problematic for two reasons. First, there is a risk that such small differences in the high and low set points cannot faithfully be achieved due to the error in hitting target expression levels (Bonde et al., 2016; Salis et al., 2009). Second, even if the small differences in set point can be achieved, an important system variable could be falsely concluded to be unimportant simply because the range of set states in that dimension was too focused.

### Blocking

Blocking is a strategy that helps minimize the impact of confounding variables and increases the precision of the experimental results (Lorenzen, 1984). Blocking involves grouping experimental units into homogeneous blocks based on levels of blocking factors, or also called nuisance factors, which are known or suspected to affect the response variable but are not of your primary interest. Blocks are created as homogeneously as possible with respect to the blocking factors, so that any variability within each block is minimized. Within each block, the assignment of treatments or experimental conditions is randomized to ensure that any systematic differences between treatments are not confounded with the blocking factors. Randomization helps ensure that the effects of the blocking factors are evenly distributed across the different treatment groups. Each treatment combination is often replicated within each block to increase the precision and reliability of the estimates of treatment effects. After data collection, statistical analysis techniques such as ANOVA or regression analysis are used to analyze the data and determine the significance of the treatment effects while accounting for the blocking factors (Jensen et al., 2018). Blocking factors are typically included in the analysis as covariates or factors to control their effects on the response variable.

Blocking has advantages due to its ability to reduce library size and increase the number of iterations through grouping. Generally, when running DoE optimization algorithms, there is a choice between utilizing large libraries with low iterations and utilizing small libraries with many iterations, depending on the design approach. If experiments are designed with a large sample size and a low number of experiments, such as with full factorial designs, it allows for comprehensive exploration of interactions among factors by testing various combinations. However, creating and managing a large library can be resource intensive. On the other hand, small libraries and high iterations may limit the optimization range since small libraries restrict the exploration of diverse effect spaces? Nonetheless, this approach offers benefits in terms of detailed refinement through a high number of iterations and time efficiency, as small blocks can be executed concurrently.

### Model-Based DoE for Nonlinear Systems

In classical DoE, linear interactions between variables are assumed. However, nonlinearity may arise from any unpredicted interactions among variables or with unconsidered external factors, particularly in bioprocesses. Replacing the assumption of linear interactions between the independent and dependent variables with a model for nonlinear behavior enables the implementation of model-based DoE (MBDoE), also known as optimal experimental design. MBDoE can outperform classical DoE but only if the model used to describe the system is appropriate. Software tools exist for using MBDoE to explore nonlinear systems in which uncertainty is mitigated using quasi– or pseudo–Monte Carlo modeling, such as the GNU Scientific Library, libseq, the DAKOTA (Design Analysis Kit for Optimization and Terascale Applications) Toolkit, and so on (Friedel & Keller, 2002; Galassi et al., 2013; Giunta et al., 2003). We are not endorsing a particular product and do not have experience using each of these.

In an implementation of MBDoE, Krausch et al. (2019) have reported that Monte Carlo sampling can provide a better insight into the probability distribution of parameter estimates for highly nonlinear models compared to traditional linearization methods and should be utilized to assess the reliability of the approximations made. They have also proposed a robust method to find optimal experimental designs using Monte Carlo simulation (Krausch et al., 2019). Monte Carlo modeling better reflects the diversity of experimental conditions by using distinct probability distributions for each input variable, allowing exploration of interactions and nonlinear effects with diverse combinations of input variables.

### Choosing the Appropriate Experimental Design

Simulation experiments have demonstrated the sensitivity of DoE methods to the degree of variable codependence (Hsu et al., 2023), or what Wright describes as “landscape ruggedness” (Wright, 1932). The ruggedness of a landscape can be quantified using an autocorrelation analysis of the multidimensional variable space (Kauffman & Levin, 1987). Briefly, autocorrelation measures the local variance in the dependent variable (the “height” in the landscape) as a function of the distance between points. A landscape with high autocorrelation indicates that neighboring points in the design space are very similar to each other, suggesting a smooth landscape. Conversely, a landscape with low autocorrelation indicates greater dissimilarity between neighboring points, indicating a more rugged and complex landscape. Ruggedness can alternatively be measured via Moran's I, or Geary's C approach (Chen, 2021). Understanding the landscape ruggedness is important for selecting the appropriate optimization method (Heinsch et al., 2018).

## Conclusion

Sorting through the large combinatorial design space when optimizing the performance of an engineered genetic system can be challenging. OFAT has merits, but DoE optimization strategies have the potential to be much more efficient for complex systems. The use of DoE in diverse engineering fields highlights its effectiveness. Several recent studies have explored its use in genetic system optimization.

Challenges in applying DoE for genetic optimization include nonindependence of component variables, relatively large measurement variance/noise, and imperfect ability to set quantitative variables to a precise and accurate value. However, rational design of genetic systems with quantitative control over gene expression levels has improved greatly in the past 20 years. Continued improvements will make DoE more feasible in this regard.

With more experimentation in applying DoE for genetic design optimization, it is likely that some approaches will emerge as front-runners. It will be important to determine what are the specific measurable system attributes that predict the success or failure of a given optimization approach. DoE is fruitfully applied in some areas of biology, such as the improvement of growth medium and pharmaceutical drug development. These underscore its potential impact in the field of genetic optimization. In sum, DoE is a validated approach for optimizing multivariate systems. Learning how to best integrate it to accelerate the engineering of complex genetic systems is an emerging area of synthetic biology that could benefit the whole field.
